# Total syntheses of oxygenated brazanquinones via regioselective homologous anionic Fries rearrangement of benzylic *O*-carbamates

**DOI:** 10.1186/1860-5397-2-1

**Published:** 2006-02-21

**Authors:** Glaucia Barbosa Candido Alves Slana, Mariângela Soares de Azevedo, Rosângela Sabattini Capella Lopes, Cláudio Cerqueira Lopes, Jari Nobrega Cardoso

**Affiliations:** 1 Instituto de Química, Universidade Federal do Rio de Janeiro, CT, Bl A/508, 21949-900 Rio de Janeiro – RJ, Brazil

## Abstract

Using new variations of anionic aromatic chemistry, the total synthesis of oxygenated brazanquinones (**1a-1c**), derived from β-brasan, a natural product isolated from *Caesalpina echinata*, via carbamates **2a-2c** is described.

## Introduction

The search for new synthetic routes for the total synthesis of biologically active natural products has been growing in recent years, often stimulated by the lack of synthetic drugs for the cure of diseases.

In various biological tests, several natural and synthetic brazanquinones have shown high biological activity. Cheng [1–2] in his work, evaluated (*in vitro*) a series of brazanquinones and their inhibitory activity against a series of cancer cell lines. Their activity has been attributed to their structure, in which the two ring systems are coplanar, and has attracted considerable attention as interesting synthetic targets. [3–11] However, the routes described in the literature do not offer the possibility of facile syntheses of other oxygenated analogues.

In this present work, the versatility of a new variation of anionic aromatic chemistry developed in our research group [12] will be applied in the context of rapid and efficient construction of these bioactive compounds for future biological evaluation.

**Figure 1:**
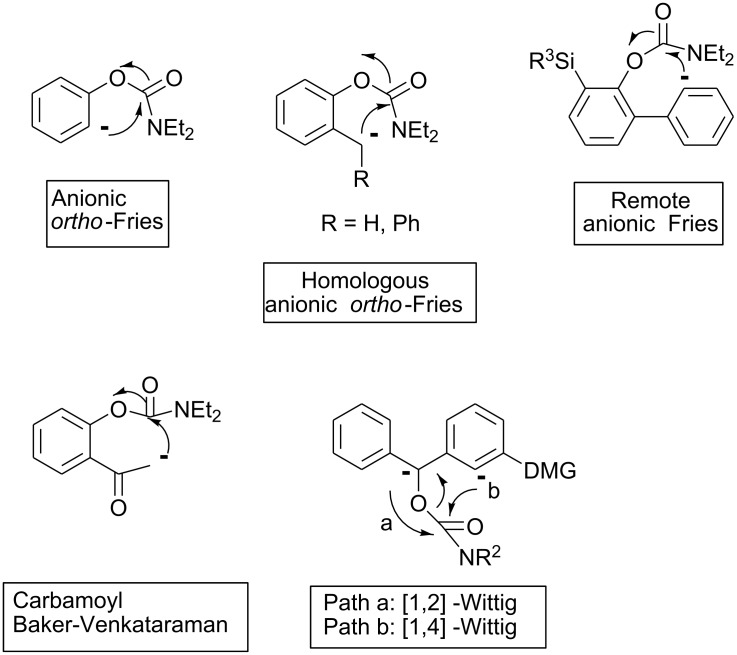
Examples of carbanionic aromatic chemistry rearrangements.

The emerging carbanionic aromatic chemistry (anionic *ortho*-Fries, [13] homologous anionic Fries, [14] remote anionic Fries rearrangements [15] and carbamoyl Baker-Venkataraman reaction [16]) originating from the Directed *ortho* Metalation (D*o*M) strategy, [17] offer a mild and regioselective complement to classical Friedel-Crafts approaches for the rational construction of polysubstituted aromatics, biaryls, and several classes of heterocycles (Figure 1).

In 1993, Gawley showed that *O*-benzylcarbamates in the presence of directed metalation groups (DMGs) undergo competitive anionic [1,2] and [1,4] Wittig – carbamoyl rearrangements (paths a and b) [18–19] orientated by the groups R and DMGs (Figure 1).

**Scheme 1:**
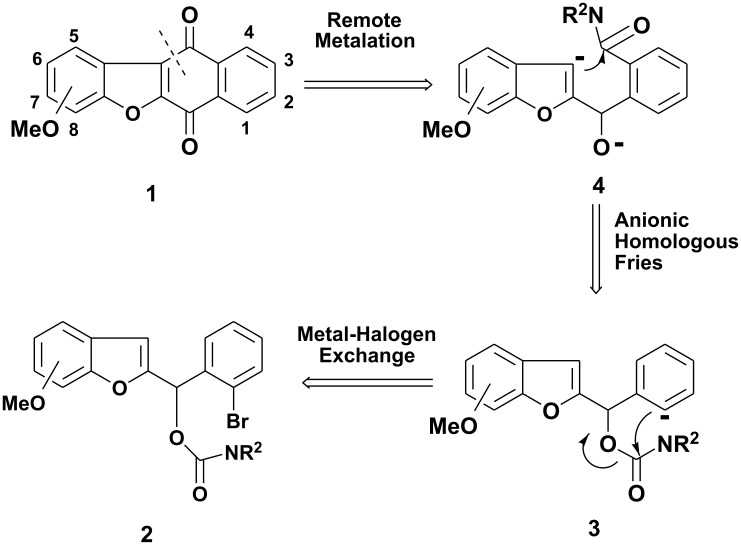
Retrosyntheses of Brazanquinones (**1**).

Conceptual combination of path b and the well established tandem D*o*M route to anthraquinones and heteroanthraquinones [20] led to the conjecture that, barring the competitive [1,2]–Wittig rearrangement, and one-pot route, **3 → 4 → 1**, may be established in a direct manner without resort to D*o*M-derived benzamide intermediate, thereby establishing new carbonyl dianion equivalency **4** (see Scheme 1).

**Scheme 2:**
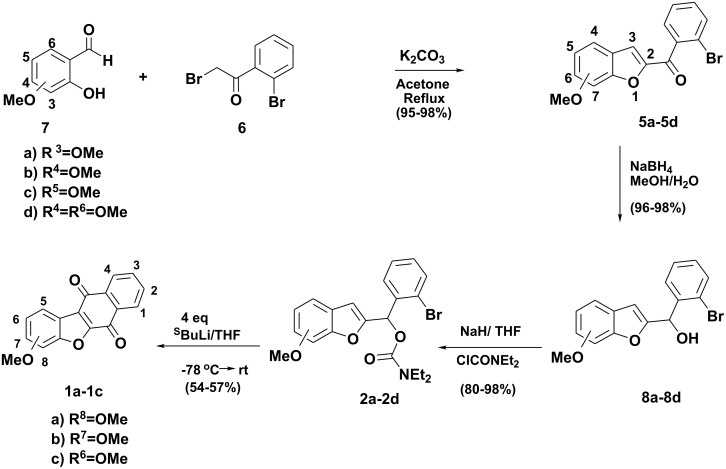
Total syntheses of brazanquinones (**1 a-c**).

We now report the versatility of this strategy, in the context of rapid and efficient construction of new oxygenated brazanquinones (**1a-1c**) derived from β-brasan, a natural product isolated from *Caesalpina echinata* (Scheme 2).

## Results and discussion

The synthesis of brazanquinones (**1a-1c**) is summarized in Scheme 2. According to our recent report, [12] the benzoylbenzofurans **5a-5d**, were prepared in one-pot reaction from α-bromoacetophenone **6** and *ortho*-hydroxybenzaldehydes **7a-7d** in excellent yields (90–96%). Reduction of benzoylbenzofurans **5a-5d** in the presence of NaBH_4_ yielded the secondary alcohols **8a-8d**, which upon treatment with *N,N*-diethylcarbamoyl chloride, and sodium hydride afforded the carbamates **2a-2d** (Scheme 2).

The carbamates **2a-2c** were cyclized in the presence of excess *sec-*BuLi in THF at -78°C to afford the desired brazanquinones **1a-1c** in reasonable yields (54%–57%) (Scheme 2).

The mechanism suggested by us and shown in Scheme 1, involves first the preparation of aryllithium intermediates **3** from the carbamates **2a-2b** by the reversible metathesis reaction known [21–23] as the lithium-halogen exchange which has been widely employed for replacement of a bromine or iodine atom in a substrate by lithium. The intermediate **3** then undergoes an intramolecular anionic Fries rearrangement to intermediate **4**, that was isolated in our previous results. [12]

Snieckus reported the remote metalation and cyclization of diethyl *N*-methyl-*O*-tolylanthranilamide to *N*-methyl dibenzazepinone [24–26] developing a new regiospecific construction of condensed aromatics. We found this route very attractive and envisaged that by *in situ* treatment of intermediate **4** with the third equivalent of *sec-*BuLi, the cyclization to the desired quinone **1a-d** would be obtained.

**Scheme 3:**
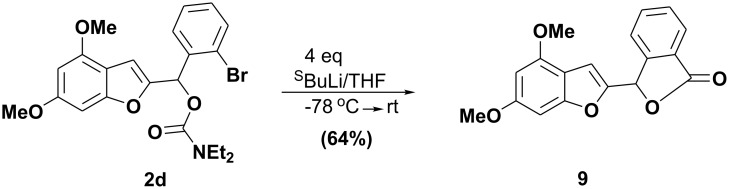
Total syntheses of phthalide (**9**).

One unexpected result was the formation of the phthalide **9** when the dimethoxy carbamate **2d** was treated with excess *sec-*BuLi under the same reaction conditions as **2a-2c**. Presumably, the presence of 4-OMe inhibits the cyclization by shielding the remote position (Scheme 3).

## Conclusion

An efficient synthesis of brazanquinones (**1a-c**) using new variations of anionic aromatic chemistry was described. This methodology could be expanded in the future for the construction of new molecules with similar structures.

## Supporting Information

File 1 contains the experimental section
