# Supplementary material for: Total syntheses of oxygenated brazanquinones via regioselective homologous anionic Fries rearrangement of benzylic O-carbamates
Source: Beilstein J Org Chem. 2006 Feb 21;2:1. doi: 10.1186/1860-5397-2-1 (PMC1408083; doi:10.1186/1860-5397-2-1)
Supplement: File 1 — contains the experimental section [file Beilstein_J_Org_Chem-02-01-s001.doc]

**Experimental**

**General information:** Melting points were determined using a Fisher hot stage apparatus and are uncorrected. ^1^H and ^13^C NMR spectra were obtained using a Bruker AC-200, AV-300 instrument in CDCl_3_ or DMSO solutions. Chemical shifts (δ) are given in parts per million (ppm) downfield relative to tetramethylsilane (TMS, δ_TMS_=0) and coupling constants (*J*) in Hz. When peak multiplicities are given, the following abbreviations are used: s (singlet), d (doublet), t (triplet), q (quartet), dd (double doublet) and m (multiplet). ^13^C NMR spectra were usually obtained using the JMOD pulse sequence. Infrared spectra were obtained using a BOMEM MB-100, MB-120 or MB-Series FTIR spectrometer, usually as liquid films or as KBr discs. Low resolution mass spectra were obtained using either a Varian Saturn GC/MS or VG 70 mass spectrometer. High resolution mass spectral analyses were performed by Universities of Ottawa or University of Toronto mass spectrometry facilities. TLC was carried out using percolated sheets (silica 60). Flash chromatography was carried out using silica gel 60 F according to Still and colleagues.^27^ Temperatures given generally refer to cooling bath temperatures and are as follows: -78^o^ C (acetone/dry ice), 0^o^ C (ice/water).

Tetrahydrofuran (THF) was freshly distilled from sodium benzophenone ketyl before use Diisopropylamine was freshly distilled from CaH_2_ before use. *N*,*N*-Diethylcarbamoyl chloride was distilled from CaH_2_ and stored under argon. Acetone was dried over K_2_CO_3_ and distilled.

Solutions of ^n^BuLi and ^S^BuLi were purchased from Aldrich Chemical Company as solutions in hexanes and cyclohexane, respectively, and regularly titrated against a standard solution of ^S^butanol with 1,10-phenanthroline as indicator.^28^

**(2-bromo-phenyl)-(7-methoxy-benzofuran-2-yl)-methanone** (**5a**)**; General procedure.** A solution of 2-methoxy-*ortho*-hydroxybenzaldehyde **7a** (0.15 g, 1.0 mL, 1.0 mmol) in 10 mL of dry acetone was added to a stirred suspension containing K_2_CO_3_ (0.14g, 1.0 mmol) in 10 mL of dry acetone. This mixture was stirred at room temperature for 15 min, and α−bromoacetophenone **6** (0.2 mg, 1.0 mmol) in 10 mL of acetone was added. The resulting mixture was stirred under reflux for 4 h and then treated with brine. The aqueous layer was extracted with EtOAc (3 x 10 mL). The organic layers were dried (Na_2_SO_4_) and concentrated. The product was purified by flash chromatography (5-10% ethyl acetate/hexane), to obtain the **(2-bromo-phenyl)-(7-methoxy-benzofuran-2-yl)-methanone 5a:** (0.34 g, 95%); salmon oil; IR *v*_max_/cm^-1^ 1660 (C=O) (KBr); ^1^H NMR (CDCl_3_, 200MHz,) *δ*_H_ 7.5 (d, *J* 10.0 Hz, 1H), 7.7–7.4 (m, 7H), 3.8 (s, 3H, OC**H_3_**); ^13^C NMR (CDCl_3_, 50 MHz) *δ*_C_ 184.8 (**C**O), 145.1, 139.2, 133.6, 131.9, 129.5, 127.3, 124.9, 121.6, 118.0, 115.3, 110.4, 56.2 (O**C**H_3_);  HRMS (M^+^) Found: 329.9894. Calc. for C_16_H_11_O_3_Br: 329.9892.

**(2-bromo-phenyl)-(6-methoxy-benzofuran-2-yl)-methanone** (**5b**)**:** From **7b** (0.15 g, 1.0 mmol); yield: (0.34 g, 96%); yellow solid, m.p. 82^o^ C (ethylacetate/dichloromethane); IR *v*_max_/cm^-1^ 1665 (C=O) (KBr); ^1^H NMR (CDCl_3_, 200MHz) *δ*_H_ 7.7–7.4 (m, 5H), 7.2 (s, 1H), 7.1 (s, 1H), 6.9 (dd, *J* 6.0 and 3.0 Hz, 1H), 3.9 (s, 3H, OC**H_3_**); ^13^C NMR (CDCl_3_, 50 MHz,) δ 183.9 (**C**O), 161.8, 158.3, 151.4, 139.7, 133.6, 131.8, 129.3, 127.2, 124.0, 120.5, 120.1, 118.9, 114.9, 95.7, 55.9 (O**C**H_3_); HRMS (M^+^) Found: 329.9889. Calc. for C_16_H_11_O_3_Br: 329.9892.

**(2-bromo-phenyl)-(5-methoxy-benzofuran-2-yl)-methanone** (**5c**)**:**  From **7c** (0.15 g, 1.0 mmol); yield: (0.34 g, 95%); orange solid, m.p. 76^o^ C (ethylacetate/dichloromethane) IR *v*_max_/cm^-1^ 1660 (C=O) (KBr); ^1^H NMR (CDCl_3_, 300MHz) *δ*_H_ 7.7 (dd, *J* 2.7 and 12.0 Hz, 1H), 7.5–7.4 (m, 5H), 7.1 (m, 2H), 3.8 (s, 3H, OC**H_3_**); ^13^C NMR (CDCl_3_, 50 MHz,) *δ*_H_ 184.8 (**C**=O), 157.1, 152.6, 152.0, 139.7, 133.9, 132.2, 129.7, 127.9, 127.6, 120.3, 119.7, 118.4, 113.7, 104.4, 56.2 (O**C**H_3_);   HRMS (M^+^) Found: 329.9861. Calc. for C_16_H_11_O_3_Br: 329.9892.

**(2-bromo-phenyl)-(4,6-dimethoxy-benzofuran-2-yl)-methanone** (**5d**)**:**  From **7d** (0.18 g, 1.0 mmol); yield: (0.38 g, 98%); yellow solid, m.p. 99-100^o^ C (ethylacetate/dichlorometha-

ne); IR *v*_max_/cm^-1^ 1652 (C=O) (KBr); ^1^H NMR (CDCl_3_, 200MHz) *δ*_H_ 7.7 (s, 1H), 7.5–7.4 (m, 3H), 7.2 (s,1H), 6.7 (s, 1H), 6.3 (s, 1H), 3.9 (s, 6H, OC**H_3_**); ^13^C NMR (CDCl_3_, 50 MHz) *δ*_C_ 183.1 (**C**=O), 163.0, 159.8, 155.3, 150.0, 138.9, 133.5, 131.6, 129.3, 127.2, 120.0, 117.3, 112.0, 95.6, 88.2, 58.9 (O**C**H_3_);   HRMS (M^+^) Found: 359.9958. Calcd. for C_17_H_13_O_4_Br: 359.9997.

**7-Methoxybenzofuran-2-yl(2-bromophenyl)carbinol** (**8a**)**; General Procedure.** An aqueous solution of NaOH 10%:NaBH_4_:H_2_O (0.5 mL: 0.03g-0.77 mmoles: 1.30 mL) , was added dropwise, to a stirred solution of (2-bromo-phenyl)-(7-methoxy-benzofuran-2-yl)-methanone **5a** (0.72 g, 1.98 mmoles) in 20 mL of MeOH at rt. After complete consumption of the starting material (TLC monitoring). Treated with water was added into the mixture. The aqueous layer was extracted with EtOAc (3 x 10 mL) and the combined organic layers washed successively with brine and dried (Na_2_SO_4_). The solvent was evaporated to obtain **7- Methoxybenzofuran-2-yl(2-bromophenyl)carbinol** (**8a**)**:** (0.71 g, 98%); colorless oil; IR *v*_max_/cm^-1^ 3398 cm^-1^ (C-**OH**) (KBr); ^1^H NMR (Acetone d^6^, 300MHz) *δ*_H_ 7.7 (d, *J* 1.2 Hz, 1H), 7.5 (d, *J* 1.2 Hz, 1H), 7.4 (m, 3H), 7.2–7.0 (m, 3H), 6.8 (d, *J* 3.0 Hz, 1H), 6.2 (s, 1H, C**H**OH), 3.9 (s, 3H, OC**H_3_**); ^13^C NMR (50 MHz, CDCl_3_) *δ*_C_  145.7, 144.8, 139.7, 133.2, 130.1, 128.9, 128.2, 123.9, 123.2, 114.0, 107.0, 105.6, 69.7 (**C**HOH), 56.0 (O**CH_3_**); HRMS (M^+^) Found: 332.0024. Calc. for C_16_H_13_O_3_Br: 332.0048.

**6-Methoxybenzofuran-2-yl(2-bromophenyl)carbinol** (**8b**) From **5b** (0.70 g, 1.92 mmoles); yield: (0.65 g, 97%); colorless oil; IR *v*_max_/cm^-1^ 3411 cm^-1^ (C-**OH**) (KBr); ^1^H NMR (CDCl_3_, 200MHz) *δ*_H_ 7.7 (d, *J* 9.0 Hz, 1H), 7.6 (d, *J* 3.0 Hz, 1H), 7.4-7.2 (m, 3H), 6.9 (s, 1H), 6.6 (dd, *J* 3.0 and 9.0 Hz, 1H), 6.4 (s, 1H), 6.3 (d, *J* 6.0 Hz, 1H, C**H**OH), 3.8 (s, 3H, OC**H_3_**); ^13^C NMR (50 MHz, CDCl_3_) *δ*_C_ 139.6, 133.0, 129.8, 128.7, 127.9, 122.9, 121.5, 121.4, 112.1, 104.9, 96.2, 94.8, 69.7 (**C**HOH), 55.9 (O**C**H_3_);  HRMS (M^+^) Found: 332.0047. Calc. for C_16_H_13_O_3_Br: 332.0048.

**5-Methoxybenzofuran-2-yl(2-bromophenyl)carbinol** (**8c**) From **5c** (0.70 g, 1.92 mmoles); yield: (0.65 g, 98%); colorless oil; IR *v*_max_/cm^-1^ 3406 cm^-1^ (C-**OH**) (KBr); ^1^H NMR (CDCl_3_, 200MHz) *δ*_H_ 7.8 (d, *J* 12 Hz, 1H), 7.6 (dd, *J* 3.0 and 12.0 Hz, 1H), 7.4 (t, *J* 1.2 Hz, 1H), 7.2-7.0 (m, 2H), 6.9 (dd, *J* 3.0 and 12.0 Hz, 1H), 6.4 (s, 1H), 6.3 (s, 1H), 5.2 (s, 1H, C**H**OH), 3.8 (s, 3H, OC**H_3_**); ^13^C NMR (50 MHz, CDCl_3_) *δ*_C_  139.5, 132.9, 129.9, 128.7, 128.6, 127.9, 122.9, 119.5, 113.3, 111.9, 105.1, 103.9, 69.7 (**C**HOH), 56.1 (O**C**H_3_);  HRMS (M^+^) Found: 331.9988. Calc. for C_16_H_13_O_3_Br: 332.0048.

**4,6-Methoxybenzofuran-2-yl(2-bromophenyl)carbinol** (**8d**) From **5d** (0.78 g, 1.61 mmoles); yield: (0.63 g, 96%); colorless oil; IR *v*_max_/cm^-1^ 3410 cm^-1^ (C-**OH**) (KBr); ^1^H NMR (CDCl_3_, 200MHz) *δ*_H_ 7.7 (m, 1H), 7.5 (d, *J* 1.2 Hz, 1H), 7.4 (t, *J* 9.0 Hz, 1H), 7.2 (m, 2H), 6.6 (s, 1H), 6.4 (s, 1H), 6.3 (s, 2H), 3.8 (s, 6H, OC**H_3_**); ^13^C NMR (50 MHz, CDCl_3_) *δ*_C_  139.6, 132.8, 129.7, 128.6, 127.8, 122.6, 102.5, 94.4, 88.4, 69.5 (**C**HOH), 55.9 (O**C**H_3_), 55.6 (O**C**H_3_);  HRMS (M^+^) Found: 362.0131. Calc. for C_17_H_15_O_4_Br: 362.0154.

**α-(2-bromophenyl)-7-methoxybenzofuran-2-ylmethyl-*N-N-*diethylcarbamate** (**2a**)**;**

**General procedure.** To a suspension of NaH 60% (6.22 mmoles, 0.245 g) in 10 mL of THF, at 0^o^ C, 7-Methoxybenzofuran-2-yl(2-bromophenyl)carbinol, **8a** (6.22 mmoles, 2.27 g) in 50mL of THF was added dropwise. The mixture was stirred at room temperature for 2h under argon atmosphere, and *N,N*-diethylcarbamoyl chloride (3.9 mL, 6.84 mmoles) was added. The reaction was stirred for 1 h and the resulting mixture was acidified with 10 mL of HCl 5%, and extracted with EtOAc (3 x 5 mL). The combined organic layers were washed with brine and dried (Na_2_SO_4_). The solvent was evaporated to obtain α-**(2-bromophenyl)-7-methoxybenzofuran-2-ylmethyl-*N-N*-diethylcarbamate 2a:** (2.80 g, 97%); orange solid, m.p.116–117^o^ C (ethylacetate/hexanes); IR *v*_max_/cm^-1^ 1706 cm^-1^ (C=O) (KBr); ^1^H NMR (CDCl_3_, 300MHz) *δ*_H_ 7.5 (dd, *J* 6.0 and 9.0 Hz, 1H), 7.4–7.0 (m, 5H), 6.9 (s, 1H, C**H**OH), 6.8 (d, *J* 3.0 Hz, 1H), 6.4 (s, 1H), 3.8 (s, 3H, OC**H_3_**), 3.3–4.0 (m, 4H, N(C**H_2_**)_2_R), 1.0 (m, 6H, NR(C**H_3_**)_2_); ^13^C NMR (50 MHz, CDCl_3_) *δ*_C_  156.6, 154.9 (**C**=O), 145.7, 137.9, 133.4, 130.1, 128.9, 128.0, 123.9, 123.4, 113.9, 107.6, 107.3, 71.4 (**C**HOH), 56.6 (O**C**H_3_), 42.5 (N(CH_2_)_2_R), 41.8 (N(**C**H_2_)_2_R), 13.2 (NR(**C**H_3_)_2_); HRMS (M^+^) Found: 431.0735. Calc. for C_21_H_22_NO_4_Br: 431.0732 .

α-**(2-bromophenyl)-6-methoxybenzofuran-2-ylmethyl-*N-N*-diethylcarbamate 2b:** From **8b** (2.22g, 6.08 mmoles,); yield: (2.71 g, 96%); orange solid, m.p. 112–113^o^ C (ethylacetate/hexanes); IR *v*_max_/cm^-1^ 1700 cm^-1^ (C=O) (KBr); ^1^H NMR (CDCl_3_, 300MHz) *δ*_H_  7.6 (t, *J* 3.0 Hz, 2H), 7.5–7.4 (m, 3H), 7.2 (s, 1H), 6.8 (d, *J* 3.0 Hz, 2H), 6.5 (s, 1H), 3.9 (s, 3H, OC**H_3_**), 3.3–3.0 (m, 4H, N(C**H_2_**)_2_R), 1.0 (m, 6H, NR(C**H_3_**)_2_); ^13^C NMR (50 MHz, CDCl_3_) *δ*_c_  158.6, 156.7 (**C**=O), 153.7, 137.8, 133.4, 130.0, 128.9, 125.9, 123.2, 121.8, 121.5, 112.4, 106.7, 104.4, 104.0, 96.4, 71.3 (**C**HOH), 56.3 (O**C**H_3_), 42.1 (N(**C**H_2_)_2_R), 41.8 (N(**C**H_2_)_2_R), 14.6 (NR(**C**H_3_)_2_, 14.2 (NR(**C**H_3_)_2_;   HRMS (M^+^) Found: 431.0739. Calc. for C_21_H_22_NO_4_Br: 431.0732.

α-**(2-bromophenyl)-5-methoxybenzofuran-2-ylmethyl-*N-N*-diethylcarbamate 2c**: From **8c** (2.27g, 6.22 mmoles); yield: 2.83 g (98%); orange solid, m.p. 116^o^ C (ethylacetate/hexanes); IR *v*_max_/cm^-1^ 1706 cm^-1^ (C=O) (KBr); ^1^H NMR (CDCl_3_, 300MHz) *δ*_H_  7.6 (dd, *J* 9.0 and 15.0 Hz, 1H), 7.6 (s, 1H), 7.4 (t, *J* 9.0 Hz, 1H), 7.3–7.0 (m, 4H), 6.8 (m, 2H), 4.0 (s, 3H, OC**H_3_**), 3.3 (m, 4H, N(C**H_2_**)_2_R), 1.6-1.0 (m, 6H, NR(C**H_3_**)_2_); ^13^C NMR (50 MHz, CDCl_3_) *δ*_C_  154.9 (**C**=O), 145.8, 137.9, 133.4, 130.1, 128.9, 128.0, 123.9, 123.4, 113.9, 107.5, 107.3, 71.3 (**C**HOH), 56.6 (O**C**H_3_), 42.8 (N(**C**H_2_)_2_R), 13.1 (NR(**C**H_3_)_2_;  HRMS (M^+^) Found: 431.0723. Calc. for C_21_H_22_NO_4_Br: 431.0732.

α-**(2-bromophenyl)-4,6-dimethoxybenzofuran-2-y-methyl-*N-N*-diethylcarbamate** **2d:** From **8d** (2.46 g, 6.22 mmoles); yield: (2.46 g, 80%); colorless oil; IR *v*_max_/cm^-1^ (KBr): 1700 cm^-1^ (C=O); ^1^H NMR (CDCl_3_, 300MHz) *δ*_H_  7.6 (dd, *J* 12.0 and 9.0 Hz, 2H), 7.3 (m, 2H), 7.2 (m, 2H), 6.7 (s, 1H, C**H**OH), 6.5 (s, 1H), 3.8 (s, 6H, OC**H_3_**), 3.4 (s, 4H, N(C**H_2_**)_2_R), 1.0 (s, 6H, NR(C**H_3_**)_2_); ^13^C NMR (50 MHz, CDCl_3_) *δ*_C_  154.0 (**C**=O), 152.3, 137.9, 133.4, 129.9, 128.8, 127.9, 104.3, 94.6, 88.7, 71.3 (**C**HOH), 56.1 (O**C**H_3_), 42.5 (N(**C**H_2_)_2_R), 41.8 (NR(CH_3_)_2_, 14.7 (NR(**C**H_3_)_2_, 13.8 (NR(**C**H_3_)_2_; HRMS (M^+^) Found: 461.0842. Calc. for C_22_H_24_NO_5_Br: 461.0838.

**8-Methoxy-Benzo[B]naphtho[2,3-d]furan-6,11-dione.** (**1a**)**; General procedure;** A solution of ^S^ BuLi (22.4 mmoles, 9.7 mL of 2.3 M solution in hexane) was added to a cold (-78 ^o^C) solution containing α-(2-bromophenyl)-5-methoxybenzofuran-2-yl-methyl-*N-N*-diethylcarbamate **2a** (2.6 g, 5.6 mmoles) in 20 mL of THF. The resultant mixture was stirred for 1 h at 78 ^o^C and allowed to warm to room temperature over 2 h.

A solution of 30 mL of NH_4_Cl 10% was added, and the resulting mixture extracted with EtOAc (3 x 15 mL). The combined organic layers were washed successively with brine and dried (Na_2_SO_4_). The product was purified by flash chromatography (30-60% ethyl acetate/hexane) to **8-Methoxy-Benzo[B]naphtho[2,3-d]furan-6,11-dione 1a: (**0.85 g, 54%); orange solid, m.p. 173^o^ C (ethylacetate/chloroform); IR *v*_max_/cm^-1^ 1679, 1621 cm^-1^ (C=O) (KBr); ^1^H NMR (CDCl_3_, 300MHz) *δ*_H_ 8.2 (m, 2H, H_1_ and H_4_), 7.9 (d, 2H, *J* 6.0 H*_z_,* 1H, H_7_) 7.8 (m, 2H, H_2_ and H_3_), 7.4 (t, *J* 9.0 Hz, 1H, H_6_), 7.0 (t, *J* 9.0 Hz, 1H, H_5_), 3.8 (s, 3H, OC**H_3_**); ^13^C NMR (50 MHz, CDCl_3_) *δ*_C_  143.0, 137.0, 136.2, 136.0, 132.0, 128.0, 127.2, 121.2, 119.0 115.2, 114.0, 104.4, 99.2, 96.0, 56.4 (O**C**H_3_); HRMS (M^+^) Found: 278.0606. Calc. for C_17_H_10_O_4_: 278.0579.

**7-Methoxy-Benzo[B]naphtho[2,3-d]furan-6,11-dione.** **1b:** From **2b** (2.60 g, 5.6 mmoles); yield: (0.87 g, 56%); orange solid, m.p. 170^o^ C (ethylacetate/chloroform); IR *v*_max_/cm^-1^ 1680, 1623 cm^-1^ (C=O) (KBr); ^1^H NMR (CDCl_3_, 300MHz) *δ*_H_  8.3 (m, 2H, H_1_ and H_4_), 7.8 (m, 2H, H_2_ and H_3_), 7.7 (d, *J* 6.0 Hz, 1H, H_6_), 7.6 (s, 1H, H_7_), 7.5 (s, 1H, H_5_), 3.80(s, 3H, OC**H_3_**); ^13^C NMR (50 MHz, CDCl_3_) *δ*_C_  160.0 (**C**O), 134.5, 134.2, 127.3, 127.1, 120.6, 114.0, 104.9, 104.4, 58.4 (O**C**H_3_);  HRMS (M^+^) Found: 278.0579. Calc. for C_17_H_10_O_4_: 278.0579.

**6-Methoxy-Benzo[B]naphtho[2,3-d]furan-6,11-dione. 1c:** From **2c** (2.60 g, 5.6 mmoles) ;yield: (0.89 g, 57%); yellow solid, m.p. 195^o^ C (ethylacetate/chloroform); IR *v*_max_/cm^-1^ 1698, 1670 cm^-1^ (C=O) (KBr); ^1^H NMR (CDCl_3_, 300MHz) *δ*_H_  8.3 (m, 2H, H_1_ and H_4_), 7.9 (d, *J* 9.0 Hz, 1H, H_8_), 7.8 (m, 2H, H_2_ and H_3_), 7.4 (t, *J* 9.0 Hz, 1H, H_5_), 7.0 (d, 1H, H_7_), 4.1 (s, 3H, OC**H_3_**); ^13^C NMR (50 MHz, CDCl_3_) *δ*_C_  143.5, 134.5, 134.3, 132.5, 128.4, 127.3, 127.2, 121.0, 120.6, 119.8, 114.05, 104.37, 100.1, 98.6, 56.4 (O**C**H_3_); HRMS (M^+^) Found: 278.0572.Calc. for C_17_H_10_O_4_: 278.0579.

**3-(4,6-Dimethoxy-benzofuran-2-yl)-3H-isobenzofuran-1-one 9:** From **2d** (2.77g, 5.6 mmoles); yield: (1.11 g, 64%); yellow solid, m.p. 170^o^ C (ethylacetate/chloroform) ; IR *v*_max_/cm^-1^ 1770 cm^-1^ (C=O) (KBr); ^1^H NMR (CDCl_3_, 300MHz) *δ*_H_ 7.9–7.5 (m, 5H), 6.6 (s, 1H, C**H**OR), 6.6 (d, *J* 9.0 Hz, 1H, H_5_), 6.3 (s, 1H, H_7_), 3.9 (s, 6H, OC**H_3_**); ^13^C NMR (50 MHz, CDCl_3_) *δ*_C_ 158.6, 156.7, 154.9, 153.7, 137.8, 133.4, 130.0, 128.9, 127.9, 123.2, 121.8, 121.5, 112.4, 106.7, 96.5, 71.3 (**C**HOR), 56.1 (O**C**H_3_);  HRMS (M^+^) Found: 310.0997. Calc. for C_18_H_14_O_5_: 310.0842.
